# The PROMOTe study: targeting the gut microbiome with prebiotics to overcome age-related anabolic resistance: protocol for a double-blinded, randomised, placebo-controlled trial

**DOI:** 10.1186/s12877-021-02301-y

**Published:** 2021-07-01

**Authors:** Mary Ni Lochlainn, Ayrun Nessa, Alyce Sheedy, Rachel Horsfall, María Paz García, Deborah Hart, Gulsah Akdag, Darioush Yarand, Samuel Wadge, Andrei-Florin Baleanu, Kevin Whelan, Claire Steves

**Affiliations:** 1 Department of Twin Research & Genetic Epidemiology, King’s College London, 3rd Floor South Wing, St Thomas’ Hospital, SE1 7EH London, UK; 2 Department of Nutritional Sciences, King’s College London, London, UK

**Keywords:** Ageing, Sarcopenia, Gut microbiome, Anabolic resistance, Muscle

## Abstract

**Background:**

Loss of skeletal muscle mass and strength occurs with increasing age and is associated with loss of function, disability, and the development of sarcopenia and frailty. Dietary protein is essential for skeletal muscle function, but older adults do not anabolise muscle in response to protein supplementation as well as younger people, so called ‘anabolic resistance’. The aetiology and molecular mechanisms for this are not understood, however the gut microbiome is known to play a key role in several of the proposed mechanisms. Thus, we hypothesise that the gut microbiome may mediate anabolic resistance and therefore represent an exciting new target for ameliorating muscle loss in older adults.

This study aims to test whether modulation of the gut microbiome using a prebiotic, in addition to protein supplementation, can improve muscle strength (as measured by chair-rise time) versus protein supplementation alone.

**Methods:**

The study is a randomised, double-blinded, placebo-controlled trial, with two parallel arms; one will receive prebiotic and protein supplementation, and the other will receive placebo (maltodextrin) and protein supplementation. Participants will be randomised as twin pairs, with one twin from each pair in each arm. Participants will be asked to take supplementation once daily for 12 weeks in addition to resistance exercises. Every participant will receive a postal box, containing their supplements, and the necessary equipment to return faecal, urine, saliva and capillary blood samples, via post. A virtual visit will be performed using online platform at the beginning and end of the study, with measures taken over video. Questionnaires, food diary and cognitive testing will be sent out via email at the beginning and end of the study.

**Discussion:**

This study aims to provide evidence for the role of the gut microbiome in anabolic resistance to dietary protein. If those who take the prebiotic and protein supplementation have a greater improvement in muscle strength compared with those who take protein supplementation alone, this would suggest that strategies to modify the gut microbiome may reduce anabolic resistance, and therefore potentially mitigate sarcopenia and frailty in older adults.

**Trial registration:**

Clinicaltrials.gov: NCT04309292. Registered on the 2nd May 2020.

**Supplementary Information:**

The online version contains supplementary material available at 10.1186/s12877-021-02301-y.

## Background

The age of populations worldwide is increasing. According to the United Nations’ World Population Prospects 2019, by 2050, 1 in 6 people in the world will be over the age of 65, up from 1 to 11 in 2019 [1]. Nearly 12 million UK residents were aged > = 65 years in mid-2017; 18.2 % of the population [2]. With this ageing population comes an increase in age-related conditions and time spent living with age-related morbidity [3]. One contributor to such morbidity is age-associated muscle loss, which is gradual and typically involves greater loss of type II muscle fibres; the main ones involved in preventing a fall [4]. In comparison to younger people, older individuals lose more muscle with bed-rest and show an attenuated response to retraining after being immobilised [5]. Skeletal muscle has a number of other important functions beyond movement, including protein metabolism [4].

In the Health ABC Study, over three years older participants in the highest protein intake quintile lost ~ 40 % less appendicular lean mass than those in the lowest quintile [6]. High protein intake is associated with reduced rehabilitation time, better cardiovascular function, and improved mortality [7]. Unfortunately, several factors can lead to reduced protein intake in older age, including social isolation, dysphagia, slower gastric emptying etc. [7].

In addition to consuming less dietary protein [8], research has shown that older adults display anabolic resistance to protein intake, a blunted responsiveness in muscle protein synthesis in older people compared with younger adults [4]. This has led to a higher recommendations for daily protein intake of 1-1.3 g/kg/day [9], compared with the current UK Reference Nutrient Intake for adults of 0.8 g/kg/day. Many older people do not meet this, with fewer than 15 % meeting this recommendation in one UK study of 256 adults aged 65–89 years [8, 10]. Within our TwinsUK dataset; 30 % of those over 60 years were below 1 g/kg/day.

The mass of our skeletal muscle is regulated by muscle protein synthesis (MPS) and muscle protein breakdown (MPB) processes. In general, MPS rates are governed by the muscle responsiveness to an anabolic stimulus, e.g. physical activity, and/or food consumption. The opposite to an anabolic stimulus is a catabolic stressor. These stressors stimulate MPB. Examples include physical inactivity, illness, and inflammation, and older people tend to have higher rates of all three of these. Anabolic resistance has a complex aetiology, involving physical inactivity and ageing physiology. Many of the postulated mechanisms involve impairments of protein metabolism, at most levels. Muscle mass typically corresponds with muscle strength [11], however this has not been vigorously tested in older adults and depends greatly on which measurements are used.

‘Gut microbiota’ refers to the bacteria, viruses, archaea, and other microbes that live in our guts. Research into its role in maintaining health is growing and evolving rapidly in recent years. As we age, the gut microbiota’s resilience is reduced, as the system becomes more vulnerable to lifestyle changes, with increased inter-individual variability and noted changed in species richness [12].

Landmark faecal transplant studies in animals have shown the ability of the gut microbiota to change physiology; for example demonstrating body composition changes in the recipient which are reflective of the donor phenotype [13]. Importantly, this shows the potential role of gut microbiota in characterising metabolic phenotypes. One animal study showed that the transfer of gut microbiota from young killifish to older killifish was able to improve ageing conditions, and extend the lifespan of the older ones [14]. Indeed, the older transplanted fish were noted to have increased ‘spontaneous exploratory behaviour’, essentially physical activity. Several different mechanisms have been proposed for anabolic resistance, and the gut microbiome plays a role in many of these. Examples include protein digestion and absorption, gut barrier function, and inflammation [7].

Modulating the microbiome in older people may be an opportunity to intervene to arrest anabolic resistance. For many older adults a rigorous exercise programme is simply not feasible, be it due to medical reasons, personal choice, or a lack of resources. Current interventions are limited to dietary recommendations, nutritional supplements, and exercise programmes. A prebiotic is a substrate that is selectively utilized by host microorganisms conferring a health benefit [15]. One previous study of the same inulin-type fructan prebiotic in older people demonstrated a reduction in exhaustion levels, improvement in handgrip strength and a reduction in overall frailty index [16, 17].

The goal of this study is to assess whether the gut microbiome modulates anabolic resistance, therefore presenting a malleable therapeutic target for prevention and reversal of muscle loss with age.

## Methods/Design

This study is referred to in shorthand as the PROMOTe study (PROtein and Muscle in Older Twins). The aim of the PROMOTe study is to test whether prebiotic supplementation in combination with protein supplementation improves muscle strength in older ambulant community dwelling individuals.

- Objective 1: carry out an interventional dietary study to test whether modulation of the gut microbiome, in addition to protein supplementation, can improve skeletal muscle function (muscle strength) versus protein supplementation alone.
- Objective 2: To deliver a comprehensive dataset, including effect sizes, on specific causal links between protein supplementation, prebiotic supplementation, changes in the gut microbiome composition, changes in the metabolomic profile and changes in skeletal muscle function of an individual.

### Design and Setting

The study will be a randomised, placebo-controlled, double-blinded, intervention study. Participants will be randomised as twin pairs, with one twin from the pair randomised to the prebiotic arm and one twin from the pair randomised to the placebo arm. Twin pairs are matched for age, sex, early environment, baseline genetic sequence, etc. thus reducing the variability between pairs of subjects, increasing the ability to detect effects. The study is community-based, with healthy volunteer twins carrying out the study remotely, sending biological samples via post, and completing all other measures during online meetings. This will enable recruitment of a population from across the United Kingdom (UK) and will remove any risk of exposure to COVID during travelling to in-person study visits, thus ensuring resilience of the study to changes in COVID restrictions.

### Study Population

Participants will be recruited from the TwinsUK database (described in [18]) and invited to attend a virtual visit using video teleconferencing. A subset will later be invited to the department in St Thomas’ Hospital, London, UK to repeat the physical performance measures. We have selected an older study population who have a lower than recommended protein intake, and who are weaker, with below average diversity of their gut microbiome. This population represents those who stand to benefit most from a dietary intervention strategy such as the protein plus prebiotic supplement examined in this trial.

#### Inclusion criteria

- Aged ≥ 60 years.
- Dietary protein intake of < 1.3 g/kg/day based upon previously collected data in the registry.
- Able to consent.
- Access to video teleconferencing on a computer, laptop, tablet or phone device.

#### Exclusion criteria

- Severe food allergy.
- Current or recent antibiotic use (preceding 3 months).
- Currently or recent use of protein or leucine supplements (preceding 3 months).
- Currently or recent use of probiotic or prebiotics (preceding 3 months).
- Current or prior history of gastrointestinal disease e.g. gastrointestinal cancer, inflammatory bowel disease, bariatric surgery, irritable bowel syndrome.
- Chronic kidney disease Stage 3 or higher (eGFR ≤ 30mls/min).
- History of any significant injury or surgery which currently affects physical functioning and ability to undertake chair stand test.
- Weight loss of ≥ 5 % of body weight in preceding 6–12 months.
- Currently involved in other intervention studies.
- Any condition or circumstance likely to interfere with the normal conduct of the study and interpretation of the results, as judged by the investigators.

As the study population are over 60 years old, it is assumed that no pregnant women will be eligible to participate.

### Study Processes

#### Participant recruitment

Participants will be selected in twin pairs, from the TwinsUK registry [18] on the basis of the following characteristics:

1. Dietary protein intake below optimal for older adults (< 1.3 g/kg/day), to target those who are most likely to experience MPS and clinical benefit. We will use existing protein intake data from the most recent food frequency questionnaire completed as part of the larger TwinsUK longitudinal study to identify these individuals.
2. Aged ≥ 60 years, representing the typical population affected by muscle ageing..

An analysis of the TwinsUK registry shows there are 1314 registered volunteers ≥ 60 years with protein intake < 1.3 g/kg/day from which to recruit.

Volunteers who are below the mean in chair-rise time and Shannon diversity in the TwinsUK registry will be preferentially recruited first, with recruitment extending to those above the mean if necessary, increasing ability to detect improvement in function. Shannon diversity is a marker of the diversity of the gut microbiome. We hypothesise that individuals with a lower-than-average Shannon diversity will show a greater response to the prebiotic intervention.

Table 1 outlines the flowchart for the PROMOTe study. Once identified as eligible using pre-existing data, participants will be invited to take part. The study will be explained via telephone, eligibility will be assessed, and online written consent taken. Written consent will be obtained from all participants. Once consent is completed, they will be invited for a virtual baseline visit. They will be sent questionnaires covering appetite, diet, quality of life, and physical activity, and will be asked to record a 3-day food diary prior to their first virtual visit. Participants will be provided with a link to carry out an online cognitive assessment (CANTAB). A postal pack will be sent out containing all the necessary items to collect samples and carry out all measures during the subsequent virtual visits.

**Table 1:** Study Flowchart

Assessment	Eligibility	Pre-baseline	Baseline	Interim	Interim	Pre-final	Final
**Week of intervention**			**1**	**3–5**	**7–9**	**11–12**	**12**
**Visit modality**		**Video call**	**Video call**	**Telephone**	**Telephone**	**Telephone**	**Video call**
Inclusion/Exclusion Criteria checks	X						
Participant information and informed consent	X		X				
Randomisation			X				
Postal box sent out		X					
3-day food diary (post/online)		X				X	
Questionnaire (post/online)		X				X	
CANTAB cognitive test (online)		X				X	
Provide supplements (post)			X				
Faecal sample (post)			X			X	
Capillary blood sample (post)			X				X
Urine sample (post)			X			X	
Saliva sample (post)			X				X
Short Physical Performance Battery			X				X
Weight (kg) – if scales available			X				X
Height (cm)			X				X
Check compliance/ adverse effects				X	X		
Participants to count leftover supplement sachets							X

### Baseline measures

Outcome measures are summarised in Box 1. Stool, urine, saliva, and capillary blood samples will be collected by the participants themselves using the sample collection kits that will be provided to them in the postal pack. An explanatory pack will be posted with the necessary apparatus to take the sample. Participants can carry this out under guidance from the researcher on the video teleconference or in their own time. Return envelopes will be provided for all samples to be posted back to the department.

Physical examination will be performed by the participant during a video call with the lead researcher. Height will be measured with a measuring tape provided in the postal pack. Participants will be asked to weight themselves if they have a weighing scales. Weighing scales can be calibrated using a standard household item, such as a tin of beans. Baseline Short Physical Performance Battery (SPPB) will be carried out remotely (this includes chair-rise time), with live instructions from a trained researcher. A dynamometer will be provided to all participants via post to measure handgrip strength, and this measurement will be taken with guidance from the trained researcher, during the video call. Any queries regarding the questionnaire, food diary, or cognitive assessment can also be addressed during the video call.

After the virtual baseline visit, participants will undergo computer generated randomisation, as twin pairs, completed by the King’s College London Clinical Trials Unit (KCTU). Participants will be allocated into two arms, either the protein supplement plus placebo (maltodextrin) or the protein supplement plus prebiotic. After randomisation the supplements will be posted to the participant. Researchers and participants will be blinded as to which group each participant is in. The KCTU will liaise directly with the company providing the supplements to ensure complete blinding of research team. A designated member of the departmental administration team will have access to the unblinded information.

### Intervention

All participants will be provided with sachets of powdered food supplement which can be stored at room temperature. Participants will be advised to take one sachet daily with a glass of water or other drink (approx. 220 ml). Dilute/squash or fruit juice can be added to the drink to add flavour. The drink can be hot or cold; participants can also add their sachet to tea, coffee or similar, whichever is their preference. All participants will be asked to take one sachet at the same time each day, for a period of 12 weeks. The supplement sachets in each arm will be indistinguishable. Participants will be asked to continue their normal diet otherwise.

All the supplements are commercially available. They will be supplied by Bonsuvan, who will arrange for indistinguishable sachets to be prepared. The sachets contain 3.32 g of protein powder and 7.5 g of either maltodextrin or a prebiotic (Darmocare Pre®). The protein supplement in all sachets, is branches-chain amino acids, consisting of L-leucine 1660mg, L-isoleucine 830mg, and L-valine 830mg.

For participants in the intervention arm, in addition to the protein, the sachet will also contain prebiotic fructo-oligosaccharide (Darmocare Pre®, Bonsuvan), which consists of inulin (min. 3.375 mg) and fructo-oligosaccharides (FOS) (min. 3.488 mg).

For participants in the placebo arm, in addition to the protein, the sachet will also contain 7.5 g of maltodextrin powder (placebo).

The supplements contain no genetically modified organisms, maize, soy, yeast, gluten, lactose, added sucrose, gelatine, animal products, preservatives, artificial colouring, flavouring or aromatic substances.

Regular contact will be made with participants by phone/email to encourage compliance and assess for any adverse effects. All participants will be encouraged to engage in regular resistance exercise at least twice per week throughout the intervention and will be provided with written advice regarding this at the beginning of the study. All participants will be asked to retain any remaining sachets at the end of the trial period to objectively record compliance.

### End of study measures

At the end of 12 weeks, participants will be asked to collect stool, urine, saliva, and capillary blood samples again using the sample collection kits that will be provided to them in their postal pack. Measurements, and SPPB assessment will be repeated. In advance of the final visit, participants will be asked to complete another 3-day food diary, cognitive test, and to fill in another questionnaire, online. Participants will be asked to count their remaining sachets to help ascertain compliance.

If participants need more flexibility on the date of their final visit, more sachets can be posted to them to continue the intervention until their final virtual visit, to a maximum of one month’s additional intervention time.

**Primary outcome**: change in chair rise time [19].

**Secondary outcomes**:

Grip strength (kg).

Short physical performance battery [20].

Frailty index [21].

Changes in cognition (as measured by CANTAB battery) [22].

Changes in appetite (as measures by SNAQ questionnaire) [23].

International physical activity questionnaire (IPAQ) [24].

Gut microbiome: 16 S rRNA sequencing.

Serum metabolites: nuclear magnetic resonance spectroscopy.

Salivary microbiome: 16 S rRNA sequencing.

Urinary microbiome: 16 S rRNA sequencing.

### Validation measures for subset of study population

COVID-19 pandemic rules allowing, a subset of the study population will be invited to take part in an in-person visit to the department for repeat measures of those taken in the virtual visits. This will include physical measures, anthropometric measures, and biological samples. Questionnaires will not be repeated a third time. Participants will be invited as twin pairs. Those selected at random will be contacted by the research team to arrange an appointment. The visit will be arranged to take place close to the final virtual visit, in order to provide accurate validation measures for those measures taken remotely. This part of the study may not be feasible if travel restrictions and lockdown measures remain in place and will only be done if safe to do so. Participation in this validation cohort is optional.

### Study procedures

#### Consenting Process

Potential volunteers will receive a phone call detailing what the study involves. Once they have stated their interest to participate, the administrative assistant will send the volunteer information sheet and consent form to the individual either by post or email. After reading the information sheet they can ask questions by ringing in or emailing, and then decide whether to continue.

 If the participant and their twin are keen to proceed and take part in the study, they will be sent a link for an online consent form to sign. Twins who have consented will be enrolled into the study by a trained member of the administration and data management team. They will then book an appointment for a virtual visit and send out the postal pack.

Participants will not be paid for taking part in the study; however, all travel and hotel accommodation expenses will be reimbursed for any participants who attend the department for validation measures.

#### Screening Procedures

Prospective participants will undergo a thorough screening questionnaire administered over the phone by a trained researcher. Once considered eligible, participants will be booked in for their baseline virtual visit. All patients that undergo screening will be logged into a screening log associated with the study by a trained researcher part of the data management team at the Department of Twin Research, King’s College London.

#### Randomisation Procedures

Participants will undergo computer generated block randomisation in twin pairs by the King’s College London Clinical Trials Unit (KCTU). A web-based randomisation system will be designed using the bespoke KCTU randomisation system. The randomisation system will be created in collaboration with the trial analysts and the lead researchers and hosted on a dedicated server within King’s and maintained by the KCTU for the duration of the project. Researchers and participants will be blinded as to which arm each participant is in.

Participant initials and date of birth will be entered on the randomisation system, NHS number, email addresses, participant names and addresses and full postcodes will not be entered into the randomisation system. No data will be entered onto the randomisation system unless a participant has signed a consent form to participate in the trial. Randomisation will be undertaken by authorised staff onto the randomisation system by going to www.ctu.co.uk and clicking the link to access the randomisation system. A full audit trail of data entry will be automatically date and time stamped, alongside information about the user making the entry within the system.

#### Data Recording

Data entered into the database and on which the analysis will be performed are pseudonymised, using a unique identification number. At the point of publication, the identification numbers are not published. A web based electronic data capture (EDC) system will be designed, using the InferMed Macro 4 system. The EDC will be created in collaboration with the trial analysts and the CI and maintained by the King’s Clinical Trials Unit for the duration of the project. It will be hosted on a dedicated server within KCL.

Participant initials and date of birth will be entered on the EDC, NHS number, email addressed, participant names and addresses, and full postcodes will not be entered into the EDC. No data will be entered onto the EDC system unless a participant has signed a consent form to participate in the trial. Source data will be entered by recruiting site staff. A full audit trail of data entry and any subsequent changes to entered data will be automatically date and time stamped, alongside information about the user making the entry/changes within the system.

At the end of the trial, the site PI will review all the data for each participant to verify that all the data are complete and correct. At this point, all data can be formally locked for analysis. Upon request, KCTU will provide a copy of the final exported dataset to the CI in .csv format and the CI will onward distribute as appropriate. As this is a small single-site study, there is no formal data monitoring committee. The CI and the data team at the Department of Twin Research and Genetic Epidemiology will liaise with the KCTU database team to monitor the data.

#### End of Study and Follow up Procedures

The end of study will be defined as all data collection complete and the database lock. No follow up procedures will be required for this study as the participants are healthy volunteers.

### Laboratory analysis

#### Sample collection, labelling and logging

All sample kits and all participant data will be pseudo-anonymised with a unique identifier, the samples may be barcoded to link with participants unique ID. The Chief Investigator (CI) will ensure that all data collected in the study are recorded in a timely manner according to any instructions provided and the subject numbering process will commence at the point of informed consent. All sample collection kits will contain detailed instruction on storage conditions to ensure the integrity and viability of samples. Participants will be asked to record the date and time of collection of each sample. The samples returned to the laboratory will be logged using the unique barcode and/or unique ID. A complete chain-of-custody will be maintained for all samples throughout, from point of acquisition, storage and all uses, including disposal where relevant.

#### Sample analysis

The biological samples (faeces, urine, blood and saliva) will be stored at -80 °C upon receipt and subsequently used for microbiome and metabolomics analysis. These assays will be undertaken by the study investigators or designated collaborators either within or outside of the UK, with appropriate Material Transfer Agreements in place. Any remaining sample will be stored at the department for potential use in future analysis, such as to allow bridging to other new or improved technologies.

For the microbiome analysis 16 S rRNA sequencing will be carried out on the faecal samples, or shotgun metagenomics if possible, at a reputable institution with a track record of such analyses. Microbiome composition will be analysed with respect to (1) species diversity, (2) compositional differences, and (3) differences in abundance of taxa. Analysis will be carried out using statistical programs STATA and RStudio [25]. All analysis will take a hierarchical approach; crude models assessing just the variable of interest (prebiotic supplementation), and models adjusted for key biological (age, gender, exercise etc.) and technical covariates (e.g., differences in library size) will be applied. Urine and salivary samples will be stored for future microbiota analysis. Serum will be generated from the whole blood for metabolomics profiling and analysed using nuclear magnetic resonance spectroscopy.

Differences in species diversity and richness will be compared between groups in regression analysis in crude and adjusted models. Distance matrixes characterise the inter-individual differences in microbiota composition; ordination analyses and permutational multivariate analysis of variance (PERMANOVA) will be used to understand differences in composition between each study arm. Amplicon Sequence Variants will be collapsed to family, order, and phylum levels, with hierarchical models, adjusted for each potential mediator individually, then fully adjusted.

### Assessment of safety

As this study is an intervention study, safety monitoring will focus on unanticipated events involving risks to participants, including unanticipated problems that meet the definition of a serious adverse event (SAE). Adverse events will be recorded and reported to the CI. The event will also be documented and discussed with all members of the research group in the research departmental meetings. The CI will decide when unblinding is permissible and liaise with the KCTU to arrange unblinding.

All SAEs (both related and unrelated) will be recorded and reported immediately to the CI and the sponsor. Relapse and death due to an unrelated or pre-existing condition, and hospitalisation for treatment of a pre-existing condition do not require reporting as SAEs. All SAEs should be reported to the research ethics committee (REC) within 15 days of the CI becoming aware where in the opinion of the CI, the event was: related i.e. resulted from administration of any of the research procedures, or unexpected i.e. an event that is not listed in the protocol as an expected occurrence. The SAE report form for non-CTIMPs will be used (available from National Research Ethics Service website) and will be sent to the main REC for the trial.

#### Ethical approval

This interventional study will be conducted in compliance with the principles of the Declaration of Helsinki (1996), the principles of Good Clinical Practice (GCP) and in accordance with all applicable regulatory requirements including (but not limited to) the Research Governance Framework. The study may be subject to inspection and audit by King’s College London and Guy’s and St Thomas’ NHS Foundation Trust under their remit as sponsors and by other regulatory bodies. This is to ensure adherence to Good Clinical Practice and the NHS Research Governance Framework for Health and Social Care (2nd edition). The study sponsors have reviewed and approved the study, but are not involved in study design; collection, management, analysis, and interpretation; and the writing up of the results for publication.

This protocol and all related documents have been reviewed and approved by The North Scotland Research Ethics Committee. Any amendments to approved documents or newly created documents will likewise be submitted for approval. The study has been accepted for adoption on to the National Institutes for Health Research Clinical Research Network portfolio.

 This study has been presented to the Department of Twin Research Volunteer Advisory Panel, who were supportive of it, and the documents have been reviewed by the online Volunteer Advisory Panel, another group of volunteers who review materials remotely, who were also supportive.

### Reporting and Dissemination

The results of the study will be reported and disseminated at international conferences and in peer-reviewed journals, but the participant’s identity will not be revealed. The chief investigator and co-investigators will ensure that on completion of the study, the results are analysed, documented and reported.

### Compliance and withdrawal

#### Subject compliance

Regular monitoring of compliance will be achieved by regular contact with the participants either via email, text messaging, or telephone. Compliance will be judged by completion of the end of study questionnaires and by the remaining number of sachets at the end of the study. Twin pairs will be advised not to share each other’s supplements.

#### Withdrawal / dropout of subjects

Participants can withdraw their consent, without giving any reason at any time during the study by contacting the study contacts. Similarly, the participant can withdraw their consent for the continued retention and use of their samples even after they have been collected, without giving a reason. Reason for discontinuation from the study will be asked about sensitively and recorded. If a subject withdraws consent to the use of donated biological samples, the retained samples will be disposed of/destroyed.

If a participant, who has given informed consent, loses capacity to consent during the study, the participant would be withdrawn from the study. Identifiable data or tissue already collected with consent would be retained and used in the study. No further data or tissue would be collected, or any other research procedures carried out on or in relation to the participant.

### Statistical Considerations

#### Sample size calculation

From our existing data, we have observed that chair-rise time is approximately log normal, with log10(time) having a SD of 0.126. We consider a relative reduction in average time of 20 % to be both clinically important and plausible. Based on these figures, we would need complete data on 30 subjects per group (60 in total) for 80 % power. Allowing for 20 % dropouts, we would need 70 (35 per group) recruited. Based on the numbers we plan to recruit for the study, we estimate a recruitment time of 18 months.

We have estimated a powered sample size based on other studies using chair-rise time [26–28], however we note that no study has investigated this in the context of protein and/or prebiotic supplementation and is therefore an estimate.

### Statistical analysis

Analysis will assess differences in absolute chair-stand time between the two study arms. Secondary analyses will include linear mixed effects regression models to investigate associations between microbiota composition, microbial metabolite markers, markers of appetite and other lifestyle and physiological parameters including SPPB adjusting for covariates and multiple testing. An intention to treat analysis will be carried out to address participants who do not complete the study.

## Discussion

In light of the COVID-19 global pandemic and the resulting restrictions on travel, this protocol has been adapted from its original form such that all visits will be carried out online, via video teleconferencing software. Participants will take part from home, with the study visits taking place over video teleconferencing. A subset of the study population will be invited to the department for repeat physical measures for validation purposes, which will be optional to them. The landscape of research will change drastically in the post COVID-19 era and research carried out remotely, utilising technology and innovative techniques, will become both more common and necessary. This may be especially relevant for our older population, who often have more difficulty travelling to and from a hospital to take part in a study.

## Supplementary Information

**Additional file 1:**

## Data Availability

The datasets that will be generated and analysed during this study will be made available via application to the TwinsUK data team as outlined on our website: https://twinsuk.ac.uk/access-our-data-collab/.

## Abbreviations

CI: Chief Investigator
GCP: Good Clinical Practice
IPAQ: International Physical Activity Questionnaire
KCL: King’s College London
KCTU: King’s College London Clinical Trials Unit
NHS: National Health Service
REC: Research Ethics Committee
SAE: Serious Adverse Event
SPPB: Short Physical Performance Battery
