# Supplementary material for: The PROMOTe study: targeting the gut microbiome with prebiotics to overcome age-related anabolic resistance: protocol for a double-blinded, randomised, placebo-controlled trial
Source: BMC Geriatr. 2021 Jul 1;21:407. doi: 10.1186/s12877-021-02301-y (PMC8248289; doi:10.1186/s12877-021-02301-y)
Supplement: Supplementary file 1 — Additional file 1: [file 12877_2021_2301_MOESM1_ESM.docx]

ParticipantID

VisitDate

*CONFIDENTIAL*

**
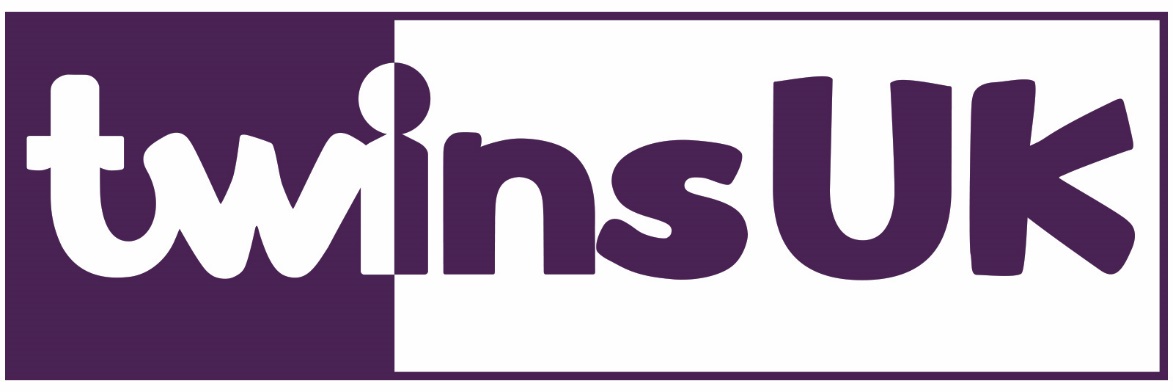
**

| **PROMOTe STudy** |
| --- |
| **For 29 years we have been a leader in health-related research.**  ***Thank you for making this happen.***  **Please continue to help our research efforts by answering the enclosed questions.** |

If you would like to receive future questionnaires online or e-newsletters, please clearly fill in your email address below ONLY if you have never given us your email address or if it has changed.

**EMAIL ADDRESS:** _ _ _ _ _ _ _ _ _ _ _ _ _ _ _ _ _ _ _ _ _ _ _ _ _ _ _ _ _ _ _ _ _ _

**QUESTIONNAIRE GUIDELINES**

1) Layout: This Questionnaire is split into topics (e.g. Behaviour).

2) Skipping: You may be instructed to skip certain questions based on your responses. Instructions on skipping are accompanied by an arrow (**🡪**).

Please, DO NOT skip ANY questions unless you are instructed to do so.

3) Estimating: You will be asked for some details that may be difficult to recall, such as the age at which you were first diagnosed with a condition. If you don’t remember the exact age at which something occurred, please give an APPROXIMATE age rather than leaving a blank – an estimate is still very helpful.

4) Response: Questions will either:

-ask you to tick the box corresponding to your answer

e.g. Have you visited any health care professional including your GP over the last two weeks?

| No | Yes |
| --- | --- |

- ask you to enter a number

e.g. How many days did you spend in hospital in total in the last year? 2   7 days

-ask you to enter a word

e.g. the name of a prescribed drug ___VENTOLIN_____

5) Please use a pen, not pencil.

**BEHAVIOUR**

The following questions are about the lifestyle choices you make such as the types of food you eat, how much you sleep and whether you drink alcohol, smoke or exercise.

*Physical Activity*

We are also interested in finding out about the kinds of physical activities that people do as part of their everyday lives. The questions will ask you about the time you spent being physically active in the **last 7 days.** Please answer each question even if you do not consider yourself to be an active person. Please think about the activities you do at work, as part of your house and yard work (gardening), to get from place to place, and in your spare time for recreation, exercise or sport.

Think about all the **vigorous** activities that you did in the **last 7 days.** Vigorous physical activities refer to activities that take hard physical effort and make you breathe much harder than normal. Think only about those physical activities that you did for at least 10 minutes at a time.

1. During the **last 7 days**, on how many days did you do vigorous physical activities like heavy lifting, aerobics, or fast bicycling?

|  | __ days per week |  |
| --- | --- | --- |
|  | No vigorous physical activity | **🡪 Go to question 3** |

1. How much time did you usually spend doing **vigorous** physical activities on one of those days?

|  | __ __ hours per day |  |
| --- | --- | --- |
|  | __ __ minutes per day |  |
|  | Don’t know/Not sure |  |

Think about all the **moderate** activities that you did in the **last 7 days. Moderate** activities refer to activities that take moderate physical effort and make you breathe somewhat harder than normal. Think only about those physical activities that you did for at least 10 minutes at a time.

1. During the **last 7 days**, on how many days did you do **moderate** physical activities like carrying light loads, bicycling at a regular pace, or doubles tennis? Do not include walking.

|  | __ days per week |  |
| --- | --- | --- |
|  | No moderate physical activity | **🡪 Go to question 5** |

1. How much time did you usually spend doing **moderate** physical activities on one of those days?

|  | __ __ hours per day |  |
| --- | --- | --- |
|  | __ __ minutes per day |  |
|  | Don’t know/Not sure |  |

Think about the time you spent **walking** in the **last 7 days**. This includes at work and at home, walking to travel from place to place, and any other walking that you have done solely for recreation, sport, exercise, or leisure.

1. During the **last 7 days**, on how many days did you **walk** for at least 10 minutes at a time?

|  | __ days per week |  |
| --- | --- | --- |
|  | No walking | **🡪 Go to question 7** |

1. How much time did you usually spend **walking** on one of those days?

|  | __ __ hours per day |  |
| --- | --- | --- |
|  | __ __ minutes per day |  |
|  | Don’t know/Not sure |  |

The next question is about the time you spent **sitting** on weekdays during the last 7 days. Include time spent at work, at home, while doing coursework and during leisure time. This may include time spent sitting at a desk, visiting friends, reading, or sitting or lying down to watch television.

1. During the **last 7 days**, how much time did you spend **sitting** on a **week day**?

|  | __ __ hours per day |  |
| --- | --- | --- |
|  | __ __ minutes per day |  |
|  | Don’t know/Not sure |  |

1. In the past year, how frequently have you typically engaged in physical exercises that raised your heart rate *and* lasted for at least 20 minutes at a time? (Note: You would know if an activity raised your heart rate since you would probably feel your heart beating faster, you would sweat, and/or feel out of breath.)

(Tick only one box)

|  | Less than once a month |
| --- | --- |
|  | At least once a month, but less than once a week |
|  | About once a week |
|  | About twice a week |
|  | - 1. times per week |
|  | 5 or more times per week |

1. During the last month, how many times have you spent more than 20 minutes swimming or cycling?

|  | Less than once a month |
| --- | --- |
|  | At least once a month, but less than once a week |
|  | About once a week |
|  | About twice a week |
|  | 3-4 times per week |
|  | 1. or more times per week |

**HEALTHCARE**

This section asks you about your use of health care services.

1. Have you visited any health care professional including your GP over the last two weeks?

| No | Yes |
| --- | --- |

1. Have you been in hospital over the last year (NOT including visits to A&E)?

| No **🡪** **Go to**  **Appetite section** | Yes**🡪** **Go to question 3** |
| --- | --- |

1. How many days did you spend in hospital in total in the last year? __ __ __ days

**NEUROLOGY, PSYCHIATRY & MENTAL HEALTH**

The branch of medicine concerning the health of the mind, brain, and nervous system.

1. During the past 12 weeks, have you experienced confusion or memory loss that is happening more often or is getting worse?

|  | No | **🡪 Go to question 19** |
| --- | --- | --- |
|  | Yes | **🡪** **Go to question 18** |

1. To what extent does memory loss currently affect your day-to-day life?

| Not at all | A little | A lot |
| --- | --- | --- |

1. During the last month, have you often been bothered by feeling down, depressed or hopeless?

| No | Yes |
| --- | --- |

1. During the last month, have you often been bothered by having little interest or pleasure in doing things?

| No | Yes |
| --- | --- |

**APPETITE**

This section asks you about your appetite.

- - - 1. My appetite is:

| Very poor  ☐ | Poor  ☐ | Average  ☐ | Good  ☐ | Very good  ☐ |
| --- | --- | --- | --- | --- |

- - - 1. When I eat

| 1. I feel full after eating only a few mouthfuls |  |
| --- | --- |
| 1. I feel full after eating about a third of a meal |  |
| 1. I feel full after eating over half of meal |  |
| 1. I feel full after eating most of the meal |  |
| 1. I hardly ever feel full |  |

- - - 1. Food tastes

| Very bad  ☐ | Bad  ☐ | Average  ☐ | Good  ☐ | Very good  ☐ |
| --- | --- | --- | --- | --- |

- - - 1. Normally I eat

| 1. Less than one meal a day |  |
| --- | --- |
| 1. One meal a day |  |
| 1. Two meals a day |  |
| 1. Three meals a day |  |
| 1. More than 3 meals a day |  |

**YOUR HEALTH & WELL-BEING**

The following questions ask for your views about your health. This information will help us to understand how you feel and how well you are able to do your usual activities.

For each of the following questions, please tick the one box that best describes your answer.

1. In general would you say your health is:

| Excellent | Very Good | Good | Fair | Poor |
| --- | --- | --- | --- | --- |

1. The following items are about activities you might do during a typical day. Does your health now limit you in these activities? If so, how much?

|  | Yes, limited a lot | Yes, limited a little | No, not limited at all |
| --- | --- | --- | --- |
| a) Moderate activities, such as moving a table, pushing a vacuum cleaner, bowling, or playing golf |  |  |  |
| b) Climbing several flights of stairs |  |  |  |

1. During the past 4 weeks, have you had any of the following problems with your work or other regular daily activities as a result of your physical health?

|  | Yes | No |
| --- | --- | --- |
| a) Accomplished less than you would like |  |  |
| b) Were limited in the kind of work or other activities |  |  |

1. During the past 4 weeks, have you had any of the following problems with your work or other regular daily activities as a result of any emotional problems (such as feeling depressed or anxious)?

|  | Yes | No |
| --- | --- | --- |
| a) Accomplished less than you would like |  |  |
| b) Didn’t do work or other activities as carefully as usual |  |  |

1. During the past 4 weeks, how much did pain interfere with your normal work (including both work outside the home and housework)?

| Not at all | A little bit | Moderately | Quite a bit | Extremely |
| --- | --- | --- | --- | --- |

1. These questions are about how you feel and how things have been with you during the past 4 weeks. For each question, please give the one answer that comes closest to the way you have been feeling. How much of the time during the past 4 weeks…

|  | All of the time | Most of the time | A good bit of the time | Some of the time | A little of the time | None of the time |
| --- | --- | --- | --- | --- | --- | --- |
| a) Have you felt calm and peaceful? |  |  |  |  |  |  |
| b) Did you have a lot of energy? |  |  |  |  |  |  |
| c) Have you felt downhearted and blue? |  |  |  |  |  |  |

1. During the past 4 weeks, how much of the time has your physical health or emotional problems interfered with your social activities (like visiting friends, relatives, etc.)?

| All of the time | Most of the time | Some of the time | A little of the time | None of the time |
| --- | --- | --- | --- | --- |

**MEDICATION HISTORY**

We would be grateful if you could provide your medication history so we can relate it to the health information you have provided in this questionnaire.

Prescription Drugs

- - - 1. Please fill in the table below with the names of your currently prescribed medication(s) including hormone treatments. “Currently prescribed” means medications/supplements/hormones that are currently taken on an intermittent or continued basis.

**Write down the prescribed drug name written on the package or bottle** **using CAPITAL LETTERS**.

a.

|  |  |  |  |  |  |  |  |  |  |  |  |  |  |  |  |  |  |  |  |  |  |  |  |  |  |  |  |  |  |  |  |  |  |  |  |  |
| --- | --- | --- | --- | --- | --- | --- | --- | --- | --- | --- | --- | --- | --- | --- | --- | --- | --- | --- | --- | --- | --- | --- | --- | --- | --- | --- | --- | --- | --- | --- | --- | --- | --- | --- | --- | --- |

b.

|  |  |  |  |  |  |  |  |  |  |  |  |  |  |  |  |  |  |  |  |  |  |  |  |  |  |  |  |  |  |  |  |  |  |  |  |  |
| --- | --- | --- | --- | --- | --- | --- | --- | --- | --- | --- | --- | --- | --- | --- | --- | --- | --- | --- | --- | --- | --- | --- | --- | --- | --- | --- | --- | --- | --- | --- | --- | --- | --- | --- | --- | --- |

c.

|  |  |  |  |  |  |  |  |  |  |  |  |  |  |  |  |  |  |  |  |  |  |  |  |  |  |  |  |  |  |  |  |  |  |  |  |  |
| --- | --- | --- | --- | --- | --- | --- | --- | --- | --- | --- | --- | --- | --- | --- | --- | --- | --- | --- | --- | --- | --- | --- | --- | --- | --- | --- | --- | --- | --- | --- | --- | --- | --- | --- | --- | --- |

d.

|  |  |  |  |  |  |  |  |  |  |  |  |  |  |  |  |  |  |  |  |  |  |  |  |  |  |  |  |  |  |  |  |  |  |  |  |  |
| --- | --- | --- | --- | --- | --- | --- | --- | --- | --- | --- | --- | --- | --- | --- | --- | --- | --- | --- | --- | --- | --- | --- | --- | --- | --- | --- | --- | --- | --- | --- | --- | --- | --- | --- | --- | --- |

e.

|  |  |  |  |  |  |  |  |  |  |  |  |  |  |  |  |  |  |  |  |  |  |  |  |  |  |  |  |  |  |  |  |  |  |  |  |  |
| --- | --- | --- | --- | --- | --- | --- | --- | --- | --- | --- | --- | --- | --- | --- | --- | --- | --- | --- | --- | --- | --- | --- | --- | --- | --- | --- | --- | --- | --- | --- | --- | --- | --- | --- | --- | --- |

f.

|  |  |  |  |  |  |  |  |  |  |  |  |  |  |  |  |  |  |  |  |  |  |  |  |  |  |  |  |  |  |  |  |  |  |  |  |  |
| --- | --- | --- | --- | --- | --- | --- | --- | --- | --- | --- | --- | --- | --- | --- | --- | --- | --- | --- | --- | --- | --- | --- | --- | --- | --- | --- | --- | --- | --- | --- | --- | --- | --- | --- | --- | --- |

g.

|  |  |  |  |  |  |  |  |  |  |  |  |  |  |  |  |  |  |  |  |  |  |  |  |  |  |  |  |  |  |  |  |  |  |  |  |  |
| --- | --- | --- | --- | --- | --- | --- | --- | --- | --- | --- | --- | --- | --- | --- | --- | --- | --- | --- | --- | --- | --- | --- | --- | --- | --- | --- | --- | --- | --- | --- | --- | --- | --- | --- | --- | --- |

h.

|  |  |  |  |  |  |  |  |  |  |  |  |  |  |  |  |  |  |  |  |  |  |  |  |  |  |  |  |  |  |  |  |  |  |  |  |  |
| --- | --- | --- | --- | --- | --- | --- | --- | --- | --- | --- | --- | --- | --- | --- | --- | --- | --- | --- | --- | --- | --- | --- | --- | --- | --- | --- | --- | --- | --- | --- | --- | --- | --- | --- | --- | --- |

i.

|  |  |  |  |  |  |  |  |  |  |  |  |  |  |  |  |  |  |  |  |  |  |  |  |  |  |  |  |  |  |  |  |  |  |  |  |  |
| --- | --- | --- | --- | --- | --- | --- | --- | --- | --- | --- | --- | --- | --- | --- | --- | --- | --- | --- | --- | --- | --- | --- | --- | --- | --- | --- | --- | --- | --- | --- | --- | --- | --- | --- | --- | --- |

j.

|  |  |  |  |  |  |  |  |  |  |  |  |  |  |  |  |  |  |  |  |  |  |  |  |  |  |  |  |  |  |  |  |  |  |  |  |  |
| --- | --- | --- | --- | --- | --- | --- | --- | --- | --- | --- | --- | --- | --- | --- | --- | --- | --- | --- | --- | --- | --- | --- | --- | --- | --- | --- | --- | --- | --- | --- | --- | --- | --- | --- | --- | --- |

|  |  |  |  |  |  |  |  |  |  |  |  |  |  |  |  |  |  |  |  |  |  |  |  |  |  |  |  |  |  |  |  |  |  |  |  |  |
| --- | --- | --- | --- | --- | --- | --- | --- | --- | --- | --- | --- | --- | --- | --- | --- | --- | --- | --- | --- | --- | --- | --- | --- | --- | --- | --- | --- | --- | --- | --- | --- | --- | --- | --- | --- | --- |

k.

(You may continue on a separate sheet if necessary. If so, please add your ParticipantID as written on the top of this page).

Non-prescription or “over the counter” drugs

- - - 1. Please fill in the table below with the names of the non-prescribed, over-the-counter medication that you are taking at least once a week on a regular basis or more than 50 times in the year. e.g. non-prescribed pain killers, aspirin, anti-histamines, cold medicine.

**Write down the prescribed drug name written on the package or bottle** **using CAPITAL LETTERS.**

a.

|  |  |  |  |  |  |  |  |  |  |  |  |  |  |  |  |  |  |  |  |  |  |  |  |  |  |  |  |  |  |  |  |  |  |  |  |  |
| --- | --- | --- | --- | --- | --- | --- | --- | --- | --- | --- | --- | --- | --- | --- | --- | --- | --- | --- | --- | --- | --- | --- | --- | --- | --- | --- | --- | --- | --- | --- | --- | --- | --- | --- | --- | --- |

b.

|  |  |  |  |  |  |  |  |  |  |  |  |  |  |  |  |  |  |  |  |  |  |  |  |  |  |  |  |  |  |  |  |  |  |  |  |  |
| --- | --- | --- | --- | --- | --- | --- | --- | --- | --- | --- | --- | --- | --- | --- | --- | --- | --- | --- | --- | --- | --- | --- | --- | --- | --- | --- | --- | --- | --- | --- | --- | --- | --- | --- | --- | --- |

c.

|  |  |  |  |  |  |  |  |  |  |  |  |  |  |  |  |  |  |  |  |  |  |  |  |  |  |  |  |  |  |  |  |  |  |  |  |  |
| --- | --- | --- | --- | --- | --- | --- | --- | --- | --- | --- | --- | --- | --- | --- | --- | --- | --- | --- | --- | --- | --- | --- | --- | --- | --- | --- | --- | --- | --- | --- | --- | --- | --- | --- | --- | --- |

d.

|  |  |  |  |  |  |  |  |  |  |  |  |  |  |  |  |  |  |  |  |  |  |  |  |  |  |  |  |  |  |  |  |  |  |  |  |  |
| --- | --- | --- | --- | --- | --- | --- | --- | --- | --- | --- | --- | --- | --- | --- | --- | --- | --- | --- | --- | --- | --- | --- | --- | --- | --- | --- | --- | --- | --- | --- | --- | --- | --- | --- | --- | --- |

e.

|  |  |  |  |  |  |  |  |  |  |  |  |  |  |  |  |  |  |  |  |  |  |  |  |  |  |  |  |  |  |  |  |  |  |  |  |  |
| --- | --- | --- | --- | --- | --- | --- | --- | --- | --- | --- | --- | --- | --- | --- | --- | --- | --- | --- | --- | --- | --- | --- | --- | --- | --- | --- | --- | --- | --- | --- | --- | --- | --- | --- | --- | --- |

f.

|  |  |  |  |  |  |  |  |  |  |  |  |  |  |  |  |  |  |  |  |  |  |  |  |  |  |  |  |  |  |  |  |  |  |  |  |  |
| --- | --- | --- | --- | --- | --- | --- | --- | --- | --- | --- | --- | --- | --- | --- | --- | --- | --- | --- | --- | --- | --- | --- | --- | --- | --- | --- | --- | --- | --- | --- | --- | --- | --- | --- | --- | --- |

g.

|  |  |  |  |  |  |  |  |  |  |  |  |  |  |  |  |  |  |  |  |  |  |  |  |  |  |  |  |  |  |  |  |  |  |  |  |  |
| --- | --- | --- | --- | --- | --- | --- | --- | --- | --- | --- | --- | --- | --- | --- | --- | --- | --- | --- | --- | --- | --- | --- | --- | --- | --- | --- | --- | --- | --- | --- | --- | --- | --- | --- | --- | --- |

h.

|  |  |  |  |  |  |  |  |  |  |  |  |  |  |  |  |  |  |  |  |  |  |  |  |  |  |  |  |  |  |  |  |  |  |  |  |  |
| --- | --- | --- | --- | --- | --- | --- | --- | --- | --- | --- | --- | --- | --- | --- | --- | --- | --- | --- | --- | --- | --- | --- | --- | --- | --- | --- | --- | --- | --- | --- | --- | --- | --- | --- | --- | --- |

i.

|  |  |  |  |  |  |  |  |  |  |  |  |  |  |  |  |  |  |  |  |  |  |  |  |  |  |  |  |  |  |  |  |  |  |  |  |  |
| --- | --- | --- | --- | --- | --- | --- | --- | --- | --- | --- | --- | --- | --- | --- | --- | --- | --- | --- | --- | --- | --- | --- | --- | --- | --- | --- | --- | --- | --- | --- | --- | --- | --- | --- | --- | --- |

j.

|  |  |  |  |  |  |  |  |  |  |  |  |  |  |  |  |  |  |  |  |  |  |  |  |  |  |  |  |  |  |  |  |  |  |  |  |  |
| --- | --- | --- | --- | --- | --- | --- | --- | --- | --- | --- | --- | --- | --- | --- | --- | --- | --- | --- | --- | --- | --- | --- | --- | --- | --- | --- | --- | --- | --- | --- | --- | --- | --- | --- | --- | --- |

(You may continue on a separate sheet if necessary. If so, please add your ParticipantID as written on the top of this page).

Nutritional or food supplements

1. Please fill in the table below with the names of the nutritional or food supplements that you are taking at least once a week on a regular basis or more than 50 times in the year. e.g. vitamins, minerals, protein powder, probiotic/fibre drinks or capsules, plant stanol or sterol drinks

**Write down the product name written on the package or bottle** **using CAPITAL LETTERS.**

a.

|  |  |  |  |  |  |  |  |  |  |  |  |  |  |  |  |  |  |  |  |  |  |  |  |  |  |  |  |  |  |  |  |  |  |  |  |  |
| --- | --- | --- | --- | --- | --- | --- | --- | --- | --- | --- | --- | --- | --- | --- | --- | --- | --- | --- | --- | --- | --- | --- | --- | --- | --- | --- | --- | --- | --- | --- | --- | --- | --- | --- | --- | --- |

b.

|  |  |  |  |  |  |  |  |  |  |  |  |  |  |  |  |  |  |  |  |  |  |  |  |  |  |  |  |  |  |  |  |  |  |  |  |  |
| --- | --- | --- | --- | --- | --- | --- | --- | --- | --- | --- | --- | --- | --- | --- | --- | --- | --- | --- | --- | --- | --- | --- | --- | --- | --- | --- | --- | --- | --- | --- | --- | --- | --- | --- | --- | --- |

c.

|  |  |  |  |  |  |  |  |  |  |  |  |  |  |  |  |  |  |  |  |  |  |  |  |  |  |  |  |  |  |  |  |  |  |  |  |  |
| --- | --- | --- | --- | --- | --- | --- | --- | --- | --- | --- | --- | --- | --- | --- | --- | --- | --- | --- | --- | --- | --- | --- | --- | --- | --- | --- | --- | --- | --- | --- | --- | --- | --- | --- | --- | --- |

d.

|  |  |  |  |  |  |  |  |  |  |  |  |  |  |  |  |  |  |  |  |  |  |  |  |  |  |  |  |  |  |  |  |  |  |  |  |  |
| --- | --- | --- | --- | --- | --- | --- | --- | --- | --- | --- | --- | --- | --- | --- | --- | --- | --- | --- | --- | --- | --- | --- | --- | --- | --- | --- | --- | --- | --- | --- | --- | --- | --- | --- | --- | --- |

e.

|  |  |  |  |  |  |  |  |  |  |  |  |  |  |  |  |  |  |  |  |  |  |  |  |  |  |  |  |  |  |  |  |  |  |  |  |  |
| --- | --- | --- | --- | --- | --- | --- | --- | --- | --- | --- | --- | --- | --- | --- | --- | --- | --- | --- | --- | --- | --- | --- | --- | --- | --- | --- | --- | --- | --- | --- | --- | --- | --- | --- | --- | --- |

f.

|  |  |  |  |  |  |  |  |  |  |  |  |  |  |  |  |  |  |  |  |  |  |  |  |  |  |  |  |  |  |  |  |  |  |  |  |  |
| --- | --- | --- | --- | --- | --- | --- | --- | --- | --- | --- | --- | --- | --- | --- | --- | --- | --- | --- | --- | --- | --- | --- | --- | --- | --- | --- | --- | --- | --- | --- | --- | --- | --- | --- | --- | --- |

g.

|  |  |  |  |  |  |  |  |  |  |  |  |  |  |  |  |  |  |  |  |  |  |  |  |  |  |  |  |  |  |  |  |  |  |  |  |  |
| --- | --- | --- | --- | --- | --- | --- | --- | --- | --- | --- | --- | --- | --- | --- | --- | --- | --- | --- | --- | --- | --- | --- | --- | --- | --- | --- | --- | --- | --- | --- | --- | --- | --- | --- | --- | --- |

h.

|  |  |  |  |  |  |  |  |  |  |  |  |  |  |  |  |  |  |  |  |  |  |  |  |  |  |  |  |  |  |  |  |  |  |  |  |  |
| --- | --- | --- | --- | --- | --- | --- | --- | --- | --- | --- | --- | --- | --- | --- | --- | --- | --- | --- | --- | --- | --- | --- | --- | --- | --- | --- | --- | --- | --- | --- | --- | --- | --- | --- | --- | --- |

i.

|  |  |  |  |  |  |  |  |  |  |  |  |  |  |  |  |  |  |  |  |  |  |  |  |  |  |  |  |  |  |  |  |  |  |  |  |  |
| --- | --- | --- | --- | --- | --- | --- | --- | --- | --- | --- | --- | --- | --- | --- | --- | --- | --- | --- | --- | --- | --- | --- | --- | --- | --- | --- | --- | --- | --- | --- | --- | --- | --- | --- | --- | --- |

j.

|  |  |  |  |  |  |  |  |  |  |  |  |  |  |  |  |  |  |  |  |  |  |  |  |  |  |  |  |  |  |  |  |  |  |  |  |  |
| --- | --- | --- | --- | --- | --- | --- | --- | --- | --- | --- | --- | --- | --- | --- | --- | --- | --- | --- | --- | --- | --- | --- | --- | --- | --- | --- | --- | --- | --- | --- | --- | --- | --- | --- | --- | --- |

(You may continue on a separate sheet if necessary. If so, please add your ParticipantID as written on the top of this page).

**MICROBIOME**

1. Have you used any antimicrobial medication i.e. antibiotics, antivirals, antifungals (not including creams or mouthwashes) in the **last 12 weeks**?

| No **→ Go to question 3** | Yes |
| --- | --- |

1. What antimicrobial medications have you used in the last 12 weeks? For each medication taken, write the reference number which can be found in the table on page 13. Give the date when you started using the medication and the duration of the course of treatment. Please give approximate dates if actual starting dates are not remembered.

(You may continue on a separate sheet if necessary. If so, please add your Participant ID as written on the top of this page).

| Reference Number | Date started (MM/YY) | Duration  (in days) |
| --- | --- | --- |
| e.g. 21 | 06/15 | 7 |
|  | __ __/__ __ | __ __ __ |
|  | __ __/__ __ | __ __ __ |
|  | __ __/__ __ | __ __ __ |
|  | __ __/__ __ | __ __ __ |
|  | __ __/__ __ | __ __ __ |
|  | __ __/__ __ | __ __ __ |
|  | __ __/__ __ | __ __ __ |

1. Have you experienced any of the following in the **last 4 weeks**?

|  | **No** | **Yes** |
| --- | --- | --- |
| 1. Diarrhoea | ☐ | ☐ |
| 1. Constipation | ☐ | ☐ |
| 1. Vomiting | ☐ | ☐ |
| 1. Cold | ☐ | ☐ |

*Your Bowel*

1. How many bowel movements have you had in the last 7 days (Tick 1 box only)?

| None | One | 2-3 | 4-6 | 7 or more |
| --- | --- | --- | --- | --- |

1. Has the frequency of your bowel movements changed in the last 12 weeks/3 months?

| No | Yes |
| --- | --- |

1. Using the Stool Chart on page 12, please define the consistency of your stool on average in the last 12 weeks/3 months.

| **Stool Type (Tick one box that best matches your stool)** | | | | | | |
| --- | --- | --- | --- | --- | --- | --- |
| Type 1  ☐ | Type 2  ☐ | Type 3  ☐ | Type 4  ☐ | Type 5  ☐ | Type 6  ☐ | Type 7  ☐ |

**Please provide your stool sample before completing the following questions**

1. At the time of providing your stool sample, did you experience the following?

|  | **No** | **Yes** |
| --- | --- | --- |
| 1. Constipation | ☐ | ☐ |
| 1. Diarrhoea | ☐ | ☐ |

1. Using the Stool Chart, please define the consistency of your stool sample provided to us.

| **Stool Type (Tick one box that best matches your sample provided)** | | | | | | |
| --- | --- | --- | --- | --- | --- | --- |
| Type 1  ☐ | Type 2  ☐ | Type 3  ☐ | Type 4  ☐ | Type 5  ☐ | Type 6  ☐ | Type 7  ☐ |


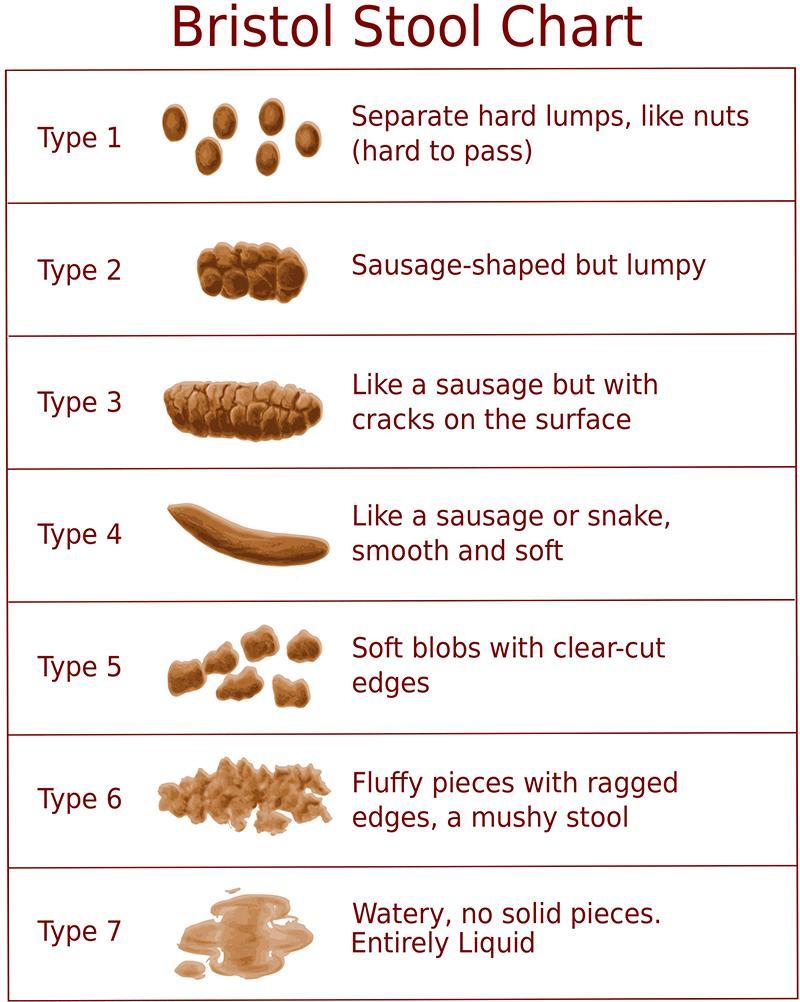


**Antimicrobial Name Table**

Antimicrobial names are listed in alphabetical order. Please ensure you are only listing those you have taken orally in the past 12 months. If the name of the medication you are looking for is not listed, please see the codes for ‘other medication’ at the bottom right of the table.

| Antimicrobial Name | Reference Number |  | Antimicrobial Name | Reference Number |
| --- | --- | --- | --- | --- |
| Aciclovir | 221 |  | Fucidin | 81 |
| Acyclovir | 221 |  | Fusidic acid | 81 |
| Amoxicillin | 1 |  | Keflex | 12 |
| Amoxicillin/clavulanate | 2 |  | Ketoconazole | 215 |
| Amoxil | 1 |  | Klaricid | 42 |
| Ampicillin | 3 |  | Lamisil | 214 |
| Ampicillin/sulbactam | 4 |  | Levofloxacin | 23 |
| Augmentin | 2 |  | Linezolid | 101 |
| Azithromycin | 47 |  | Lymecycline | 35 |
| Canesten | 212 |  | Macrodantin | 51 |
| Cefaclor | 19 |  | Methicillin | 6 |
| Cefadroxil | 11 |  | Metronidazole | 61 |
| Cefalexin | 12 |  | Miconazole | 213 |
| Cefixime | 13 |  | Minocycline | 36 |
| Cefotaxime | 14 |  | Moxifloxacin | 24 |
| Cefradine | 18 |  | Nitrofurantoin | 51 |
| Ceftazidime | 15 |  | Norfloxacin | 25 |
| Ceftriaxone | 16 |  | Ofloxacin | 22 |
| Cefuroxime | 17 |  | Oseltamivir | 201 |
| Chloramphenicol | 91 |  | Oxytetracycline | 32 |
| Cipro | 21 |  | Penicillin | 7 |
| Ciprofloxacin | 21 |  | Pfizerpen/ Penicillin G | 7 |
| Ciproxin | 21 |  | Phenoxymethyl Penicillin | 7 |
| Clarithromycin | 42 |  | Piperacillin | 8 |
| Clotrimazole | 211 |  | Piperacillin/tazobactam | 9 |
| Co-amoxiclav | 2 |  | Relenza | 202 |
| Co-trimoxazole | 72 |  | Tamiflu | 201 |
| Daktarin | 213 |  | Teicoplanin | 111 |
| Demeclocycline | 34 |  | Terbinafine | 214 |
| Diflucan | 212 |  | Tetracycline | 31 |
| Doxycycline | 33 |  | Tetralysal | 35 |
| Erythrocin | 41 |  | Trimethoprim | 71 |
| Erythromycin | 41 |  | Valacyclovir | 222 |
| Erythroped | 41 |  | Vancomycin | 112 |
| Flagyl | 61 |  | Zovirax | 221 |
| Floxin/ Ofloxacin | 22 |  | Other Antibacterial | 130 |
| Flucloxacillin | 5 |  | Other Antifungal | 240 |
| Fluconazole | 212 |  | Other Antiviral | 230 |

Thank You

We really appreciate the time and effort it has taken for you to answer this questionnaire.

Your answers will help us to discover the important genetic and environmental causes of complex diseases and ageing and how it relates to our diet and lifestyle.

**Please return this completed questionnaire in the return envelope provided.**

**Many thanks for your ongoing help and support.**

Keep in touch:

| TwinsUK | 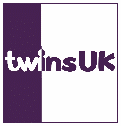 | www.twinsuk.ac.uk/ |
| --- | --- | --- |
| Facebook | 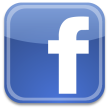 | www.facebook.com/twinsuk |
| YouTube | 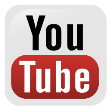 | www.youtube.com/user/DeptTwinResearch |
| Twitter | 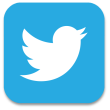 | twitter.com/twinsukres |

Contact Info:

| **Department of Twin Research & Genetic Epidemiology**  King’s College London St Thomas’ Hospital Campus 3rd & 4th Floor South Wing Block D Westminster Bridge Road London SE1 7EH  Tel for twins/general public: +44 (0) 20 7188 5555 Tel for scientific community: +44 (0) 20 7188 6765  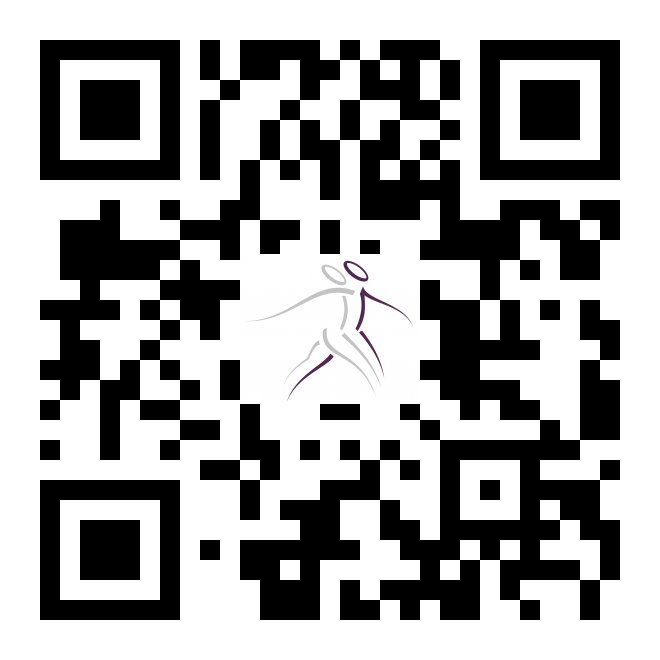 |
| --- |
